# Ethyl 1-(6-chloro-3-pyridylmeth­yl)-5-ethoxy­methyl­eneamino-1*H*-1,2,3-triazole-4-carboxyl­ate

**DOI:** 10.1107/S1600536808037197

**Published:** 2008-11-13

**Authors:** Xiao-Bao Chen, Feng-Mei Sun, Hai-Tao Gao, Jing Xu, Ai-Hua Zheng

**Affiliations:** a Department of Medicinal Chemistry, Yunyang Medical College, Shiyan 442000, People’s Republic of China; b School of Chemistry and Chemical Engineering, Henan Institute of Science and Technology, Xinxiang 453003, Henan, People’s Republic of China

## Abstract

In the title compound, C_14_H_16_ClN_5_O_3_, there is evidence for significant electron delocalization in the triazolyl system. Intra­molecular C—H⋯O and inter­molecular C—H⋯O and C—H⋯N hydrogen bonds stabilize the structure.

## Related literature

Many derivatives of triazole have been prepared, and their biological activities have been studied, see: Ogura *et al.* (2000*a* ▶,*b* ▶); Najim *et al.* (2004 ▶); Banks & Chubb (1999*a* ▶,*b* ▶); Shuto *et al.* (1995*a* ▶,*b* ▶); Yuan *et al.* (2006 ▶); Chen *et al.* (2005 ▶); Liu *et al.* (2001 ▶). For the synthesis, see: Chen & Shi (2008 ▶). For bond-length data, see: Sasada (1984 ▶); Wang *et al.* (1998 ▶).
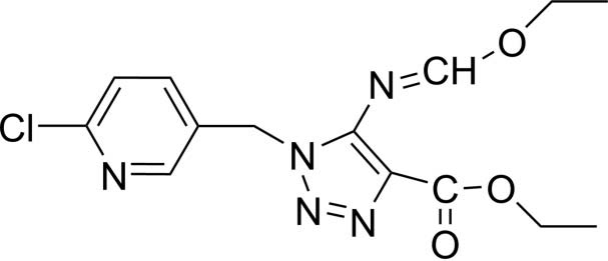


## Experimental

### 

#### Crystal data

C_14_H_16_ClN_5_O_3_
*M* *_r_* = 337.77
Monoclinic, 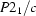

*a* = 16.8823 (17) Å
*b* = 6.3134 (6) Å
*c* = 15.3065 (15) Å
β = 90.980 (1)°
*V* = 1631.2 (3) Å^3^
*Z* = 4
Mo *K*α radiation
μ = 0.26 mm^−1^
*T* = 291 (2) K
0.50 × 0.47 × 0.36 mm

#### Data collection

Bruker SMART APEX CCD area-detector diffractometer
Absorption correction: none
10092 measured reflections
2980 independent reflections
2551 reflections with *I* > 2σ(*I*)
*R*_int_ = 0.014

#### Refinement

*R*[*F* ^2^ > 2σ(*F* ^2^)] = 0.036
*wR*(*F* ^2^) = 0.098
*S* = 1.04
2980 reflections
210 parameters
H-atom parameters constrained
Δρ_max_ = 0.25 e Å^−3^
Δρ_min_ = −0.40 e Å^−3^

### 

Data collection: *SMART* (Bruker, 2000 ▶); cell refinement: *SAINT* (Bruker, 2000 ▶); data reduction: *SAINT*; program(s) used to solve structure: *SHELXS97* (Sheldrick, 2008 ▶); program(s) used to refine structure: *SHELXL97* (Sheldrick, 2008 ▶); molecular graphics: *SHELXTL* (Sheldrick, 2008 ▶); software used to prepare material for publication: *SHELXTL*.

## Supplementary Material

Crystal structure: contains datablocks global, I. DOI: 10.1107/S1600536808037197/at2675sup1.cif

Structure factors: contains datablocks I. DOI: 10.1107/S1600536808037197/at2675Isup2.hkl

Additional supplementary materials:  crystallographic information; 3D view; checkCIF report

## Figures and Tables

**Table 1:** Hydrogen-bond geometry (Å, °)

*D*—H⋯*A*	*D*—H	H⋯*A*	*D*⋯*A*	*D*—H⋯*A*
C2—H2⋯N3^i^	0.93	2.57	3.488 (2)	167
C9—H9*A*⋯O2^ii^	0.97	2.53	3.246 (3)	131
C12—H12⋯O2	0.93	2.44	2.924 (3)	112

Symmetry codes: (i) 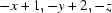; (ii) 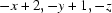.

**Fig. 1.**
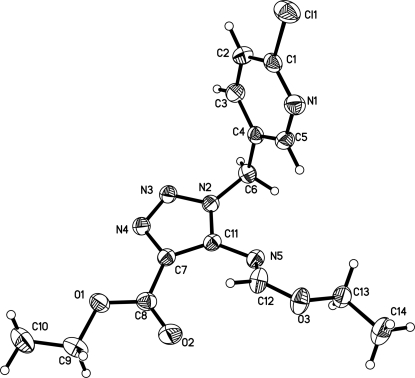
View of the molecular structure of (I), showing the atom labelling scheme and with displacement ellipsoids drawn at the 50% probability level.

**Fig. 2.**
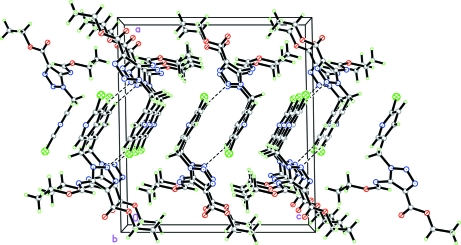
A partial view of the crystal packing of (I), showing the formation of C—H···O and C—H···N hydrogen-bonding interactions (dashed lines).

## supplementary crystallographic information

### Comment

It is well known that many triazole-related molecules play an important role in the development of agrochemicals such as insecticides, nematocides, acaricide and plant growth regulators (Ogura *et al.*, 2000*a*,*b*; Najim *et al.*, 2004; Banks & Chubb, 1999*a*,*b*); Shuto *et al.*, 1995*a*,*b*; Yuan *et al.*, 2006; Chen *et al.*, 2005 and Liu *et al.*, 2001). Since the structure-activity relationship is very useful in the rational design of pharmaceuticals and agrochemicals. We report here the crystal structure of the title compound, (I) (Fig. 1), which was synthesized by introducing pyridine rings into a 1,2,3-triazole molecular framework.

In the title compound (I), the C7—N4 and C11—N2 bonds [1.366 (2) and 1.348 (2) Å] are significantly shorter than that of the single bond of C—N (1.47 Å; Sasada, 1984) and close to the value of the double bond of C—N (1.28 Å; Wang *et al.*, 1998). This indicates significant electron delocalization in the triazolyl system. Intramolecular C—H···O and intermolecular C—H···O and C—H···N hydrogen bonds contribute strongly to the stability of the molecular configuration (Fig.2, Table 1).

### Experimental

A solution of compound 1-((6-chloropyridin-3-yl)methyl)-4-ethoxycarbonyl-5-amine-1*H*-1,2,3- triazole (2 mmol) in triethyl *ortho* formate (10 ml) was refluxed for 4 h, cooled briefly and evaporated. The residue was purified by chromatography on a silica gel column by eluting with petroleum ether/acetone (2:1, *v*/*v*) to give the title compound (yield 75%). Colourless crystals of (I) suitable for X-ray structure analysis were grown from acetone and petroleum ether (1:3, *v*/*v*).

### Refinement

H atoms bonded to C were placed at calculated positions, with C—H = 0.93–0.97 Å and refined using a riding model, with *U*_iso_(H) = 1.2*U*_eq_(C), or 1.5*U*_eq_(methyl C).

### Crystal data

**Table:**

C_14_H_16_ClN_5_O_3_	*F*_000_ = 704
*M**_r_* = 337.77	*D*_x_ = 1.375 Mg m^−^^3^
Monoclinic, *P*2_1_/*c*	Mo *K*α radiation λ = 0.71073 Å
Hall symbol: -P 2ybc	Cell parameters from 4752 reflections
*a* = 16.8823 (17) Å	θ = 2.7–27.8º
*b* = 6.3134 (6) Å	µ = 0.26 mm^−^^1^
*c* = 15.3065 (15) Å	*T* = 291 (2) K
β = 90.9800 (10)º	Block, colourless
*V* = 1631.2 (3) Å^3^	0.50 × 0.47 × 0.36 mm
*Z* = 4	

### Data collection

**Table:**

Bruker SMART APEX CCD area-detector diffractometer	2551 reflections with *I* > 2σ(*I*)
Radiation source: fine-focus sealed tube	*R*_int_ = 0.014
Monochromator: graphite	θ_max_ = 25.5º
*T* = 291(2) K	θ_min_ = 2.7º
φ and ω scans	*h* = −20→20
Absorption correction: none	*k* = −7→7
10092 measured reflections	*l* = −18→18
2980 independent reflections	

### Refinement

**Table:**

Refinement on *F*^2^	Secondary atom site location: difference Fourier map
Least-squares matrix: full	Hydrogen site location: inferred from neighbouring sites
*R*[*F*^2^ > 2σ(*F*^2^)] = 0.036	H-atom parameters constrained
*wR*(*F*^2^) = 0.098	*w* = 1/[σ^2^(*F*_o_^2^) + (0.0449*P*)^2^ + 0.5005*P*] where *P* = (*F*_o_^2^ + 2*F*_c_^2^)/3
*S* = 1.04	(Δ/σ)_max_ = 0.001
2980 reflections	Δρ_max_ = 0.25 e Å^−^^3^
210 parameters	Δρ_min_ = −0.40 e Å^−^^3^
Primary atom site location: structure-invariant direct methods	Extinction correction: none

### Special details

**Table:**

Geometry. All e.s.d.'s (except the e.s.d. in the dihedral angle between two l.s. planes)are estimated using the full covariance matrix. The cell e.s.d.'s are takeninto account individually in the estimation of e.s.d.'s in distances, anglesand torsion angles; correlations between e.s.d.'s in cell parameters are onlyused when they are defined by crystal symmetry. An approximate (isotropic)treatment of cell e.s.d.'s is used for estimating e.s.d.'s involving l.s. planes.
Refinement. Refinement of *F*^2^ against ALL reflections. The weighted *R*-factor *wR* andgoodness of fit *S* are based on *F*^2^, conventional *R*-factors *R* are basedon *F*, with *F* set to zero for negative *F*^2^. The threshold expression of*F*^2^ > σ(*F*^2^) is used only for calculating *R*-factors(gt) *etc*. and isnot relevant to the choice of reflections for refinement. *R*-factors basedon *F*^2^ are statistically about twice as large as those based on *F*, and *R*-factors based on ALL data will be even larger.

### Fractional atomic coordinates and isotropic or equivalent isotropic displacement parameters (Å^2^)

**Table:**

	*x*	*y*	*z*	*U*_iso_*/*U*_eq_	
Cl1	0.36873 (3)	0.34616 (9)	0.06142 (3)	0.07336 (19)	
O1	0.89923 (7)	0.8586 (2)	−0.04595 (8)	0.0571 (3)	
O2	0.91635 (9)	0.6353 (3)	0.06636 (11)	0.0821 (5)	
O3	0.80412 (9)	0.27638 (19)	0.22225 (8)	0.0633 (4)	
N1	0.50169 (9)	0.4258 (2)	0.14254 (10)	0.0559 (4)	
N2	0.69532 (8)	0.9067 (2)	0.13235 (8)	0.0440 (3)	
N3	0.69871 (8)	1.0541 (2)	0.06795 (9)	0.0499 (3)	
N4	0.76295 (8)	1.0162 (2)	0.02373 (9)	0.0494 (3)	
N5	0.76407 (8)	0.6142 (2)	0.19200 (8)	0.0470 (3)	
C1	0.44375 (10)	0.5167 (3)	0.09780 (10)	0.0480 (4)	
C2	0.43841 (11)	0.7289 (3)	0.07976 (12)	0.0582 (5)	
H2	0.3959	0.7842	0.0477	0.070*	
C3	0.49856 (10)	0.8571 (3)	0.11104 (12)	0.0545 (4)	
H3	0.4972	1.0021	0.1004	0.065*	
C4	0.56076 (9)	0.7699 (3)	0.15809 (10)	0.0422 (4)	
C5	0.55899 (10)	0.5546 (3)	0.17194 (12)	0.0535 (4)	
H5	0.6006	0.4946	0.2040	0.064*	
C6	0.62874 (10)	0.9037 (3)	0.19223 (11)	0.0504 (4)	
H6A	0.6103	1.0475	0.2012	0.061*	
H6B	0.6466	0.8485	0.2483	0.061*	
C7	0.80079 (9)	0.8443 (2)	0.05900 (10)	0.0443 (4)	
C8	0.87732 (10)	0.7667 (3)	0.02805 (12)	0.0515 (4)	
C9	0.97474 (11)	0.7873 (3)	−0.08109 (14)	0.0676 (5)	
H9A	0.9744	0.6348	−0.0885	0.081*	
H9B	1.0179	0.8246	−0.0414	0.081*	
C10	0.98505 (15)	0.8939 (5)	−0.16692 (17)	0.0994 (9)	
H10A	0.9433	0.8509	−0.2065	0.149*	
H10B	1.0353	0.8547	−0.1905	0.149*	
H10C	0.9832	1.0446	−0.1591	0.149*	
C11	0.75714 (9)	0.7719 (2)	0.12926 (10)	0.0424 (4)	
C12	0.79167 (12)	0.4373 (3)	0.16850 (11)	0.0580 (5)	
H12	0.8039	0.4194	0.1100	0.070*	
C13	0.78506 (12)	0.3050 (3)	0.31347 (11)	0.0584 (5)	
H13A	0.7281	0.3109	0.3205	0.070*	
H13B	0.8080	0.4356	0.3357	0.070*	
C14	0.81906 (16)	0.1190 (4)	0.36122 (14)	0.0812 (7)	
H14A	0.7993	−0.0094	0.3353	0.122*	
H14B	0.8040	0.1248	0.4214	0.122*	
H14C	0.8758	0.1219	0.3578	0.122*	

### Atomic displacement parameters (Å^2^)

**Table:**

	*U*^11^	*U*^22^	*U*^33^	*U*^12^	*U*^13^	*U*^23^
Cl1	0.0601 (3)	0.0926 (4)	0.0674 (3)	−0.0200 (3)	0.0024 (2)	−0.0204 (3)
O1	0.0478 (7)	0.0593 (7)	0.0647 (7)	0.0024 (5)	0.0108 (6)	0.0029 (6)
O2	0.0658 (9)	0.0824 (10)	0.0985 (11)	0.0239 (8)	0.0096 (8)	0.0267 (9)
O3	0.0954 (10)	0.0428 (7)	0.0519 (7)	0.0049 (6)	0.0048 (7)	0.0053 (5)
N1	0.0510 (8)	0.0480 (8)	0.0687 (9)	−0.0032 (7)	−0.0003 (7)	0.0059 (7)
N2	0.0445 (7)	0.0411 (7)	0.0464 (7)	−0.0041 (6)	−0.0016 (6)	0.0019 (6)
N3	0.0495 (8)	0.0455 (8)	0.0546 (8)	−0.0002 (6)	−0.0009 (6)	0.0080 (6)
N4	0.0477 (8)	0.0475 (8)	0.0529 (8)	−0.0023 (6)	−0.0001 (6)	0.0067 (6)
N5	0.0504 (8)	0.0442 (8)	0.0461 (7)	−0.0011 (6)	−0.0046 (6)	0.0045 (6)
C1	0.0440 (9)	0.0595 (10)	0.0409 (8)	−0.0040 (7)	0.0085 (7)	−0.0054 (7)
C2	0.0475 (10)	0.0673 (12)	0.0596 (10)	0.0089 (8)	−0.0050 (8)	0.0090 (9)
C3	0.0537 (10)	0.0448 (9)	0.0652 (11)	0.0078 (8)	0.0022 (8)	0.0091 (8)
C4	0.0437 (8)	0.0440 (8)	0.0391 (8)	0.0033 (7)	0.0078 (6)	0.0001 (7)
C5	0.0482 (9)	0.0501 (10)	0.0620 (10)	0.0016 (8)	−0.0058 (8)	0.0132 (8)
C6	0.0540 (10)	0.0485 (9)	0.0490 (9)	−0.0009 (8)	0.0061 (7)	−0.0045 (7)
C7	0.0437 (9)	0.0413 (8)	0.0476 (9)	−0.0043 (7)	−0.0045 (7)	0.0016 (7)
C8	0.0461 (9)	0.0477 (9)	0.0607 (10)	−0.0032 (7)	−0.0022 (8)	0.0003 (8)
C9	0.0483 (10)	0.0690 (12)	0.0859 (14)	0.0012 (9)	0.0135 (10)	−0.0138 (11)
C10	0.0811 (16)	0.127 (2)	0.0917 (17)	0.0149 (16)	0.0392 (14)	0.0044 (16)
C11	0.0437 (8)	0.0391 (8)	0.0441 (8)	−0.0044 (7)	−0.0070 (7)	−0.0013 (7)
C12	0.0857 (13)	0.0450 (10)	0.0434 (9)	−0.0065 (9)	0.0000 (9)	0.0018 (8)
C13	0.0672 (12)	0.0585 (11)	0.0494 (9)	0.0055 (9)	0.0033 (8)	0.0074 (8)
C14	0.1104 (18)	0.0668 (13)	0.0665 (13)	0.0154 (12)	0.0013 (12)	0.0190 (11)

### Geometric parameters (Å, °)

**Table:**

Cl1—C1	1.7462 (17)	C4—C5	1.376 (2)
O1—C8	1.331 (2)	C4—C6	1.511 (2)
O1—C9	1.463 (2)	C5—H5	0.9300
O2—C8	1.205 (2)	C6—H6A	0.9700
O3—C12	1.322 (2)	C6—H6B	0.9700
O3—C13	1.450 (2)	C7—C11	1.391 (2)
N1—C1	1.316 (2)	C7—C8	1.468 (2)
N1—C5	1.336 (2)	C9—C10	1.489 (3)
N2—C11	1.348 (2)	C9—H9A	0.9700
N2—N3	1.3579 (18)	C9—H9B	0.9700
N2—C6	1.463 (2)	C10—H10A	0.9600
N3—N4	1.3103 (19)	C10—H10B	0.9600
N4—C7	1.366 (2)	C10—H10C	0.9600
N5—C12	1.264 (2)	C12—H12	0.9300
N5—C11	1.387 (2)	C13—C14	1.493 (3)
C1—C2	1.371 (3)	C13—H13A	0.9700
C2—C3	1.378 (3)	C13—H13B	0.9700
C2—H2	0.9300	C14—H14A	0.9600
C3—C4	1.378 (2)	C14—H14B	0.9600
C3—H3	0.9300	C14—H14C	0.9600
			
C8—O1—C9	115.82 (15)	O2—C8—O1	123.82 (16)
C12—O3—C13	117.92 (14)	O2—C8—C7	123.34 (17)
C1—N1—C5	115.93 (15)	O1—C8—C7	112.83 (15)
C11—N2—N3	111.41 (13)	O1—C9—C10	107.54 (18)
C11—N2—C6	128.15 (13)	O1—C9—H9A	110.2
N3—N2—C6	120.40 (13)	C10—C9—H9A	110.2
N4—N3—N2	107.16 (13)	O1—C9—H9B	110.2
N3—N4—C7	109.08 (13)	C10—C9—H9B	110.2
C12—N5—C11	117.65 (14)	H9A—C9—H9B	108.5
N1—C1—C2	125.22 (16)	C9—C10—H10A	109.5
N1—C1—Cl1	115.20 (13)	C9—C10—H10B	109.5
C2—C1—Cl1	119.57 (14)	H10A—C10—H10B	109.5
C1—C2—C3	117.28 (16)	C9—C10—H10C	109.5
C1—C2—H2	121.4	H10A—C10—H10C	109.5
C3—C2—H2	121.4	H10B—C10—H10C	109.5
C4—C3—C2	119.84 (16)	N2—C11—N5	119.02 (14)
C4—C3—H3	120.1	N2—C11—C7	103.90 (13)
C2—C3—H3	120.1	N5—C11—C7	137.02 (15)
C5—C4—C3	117.18 (16)	N5—C12—O3	123.85 (16)
C5—C4—C6	121.16 (15)	N5—C12—H12	118.1
C3—C4—C6	121.65 (15)	O3—C12—H12	118.1
N1—C5—C4	124.54 (16)	O3—C13—C14	106.49 (16)
N1—C5—H5	117.7	O3—C13—H13A	110.4
C4—C5—H5	117.7	C14—C13—H13A	110.4
N2—C6—C4	112.19 (13)	O3—C13—H13B	110.4
N2—C6—H6A	109.2	C14—C13—H13B	110.4
C4—C6—H6A	109.2	H13A—C13—H13B	108.6
N2—C6—H6B	109.2	C13—C14—H14A	109.5
C4—C6—H6B	109.2	C13—C14—H14B	109.5
H6A—C6—H6B	107.9	H14A—C14—H14B	109.5
N4—C7—C11	108.44 (14)	C13—C14—H14C	109.5
N4—C7—C8	123.09 (14)	H14A—C14—H14C	109.5
C11—C7—C8	128.35 (15)	H14B—C14—H14C	109.5
			
C11—N2—N3—N4	−0.55 (17)	C9—O1—C8—C7	−179.47 (14)
C6—N2—N3—N4	−178.53 (13)	N4—C7—C8—O2	169.02 (17)
N2—N3—N4—C7	0.30 (17)	C11—C7—C8—O2	−6.6 (3)
C5—N1—C1—C2	0.0 (3)	N4—C7—C8—O1	−10.2 (2)
C5—N1—C1—Cl1	−179.06 (13)	C11—C7—C8—O1	174.16 (15)
N1—C1—C2—C3	0.0 (3)	C8—O1—C9—C10	175.16 (19)
Cl1—C1—C2—C3	179.07 (13)	N3—N2—C11—N5	178.28 (13)
C1—C2—C3—C4	0.2 (3)	C6—N2—C11—N5	−3.9 (2)
C2—C3—C4—C5	−0.4 (2)	N3—N2—C11—C7	0.55 (17)
C2—C3—C4—C6	178.87 (15)	C6—N2—C11—C7	178.34 (14)
C1—N1—C5—C4	−0.3 (3)	C12—N5—C11—N2	144.61 (16)
C3—C4—C5—N1	0.4 (3)	C12—N5—C11—C7	−38.6 (3)
C6—C4—C5—N1	−178.81 (16)	N4—C7—C11—N2	−0.36 (17)
C11—N2—C6—C4	−88.86 (19)	C8—C7—C11—N2	175.79 (15)
N3—N2—C6—C4	88.75 (17)	N4—C7—C11—N5	−177.45 (17)
C5—C4—C6—N2	85.12 (19)	C8—C7—C11—N5	−1.3 (3)
C3—C4—C6—N2	−94.08 (18)	C11—N5—C12—O3	177.40 (16)
N3—N4—C7—C11	0.04 (18)	C13—O3—C12—N5	−0.1 (3)
N3—N4—C7—C8	−176.36 (15)	C12—O3—C13—C14	−169.17 (18)
C9—O1—C8—O2	1.3 (3)		

### Hydrogen-bond geometry (Å, °)

**Table:**

*D*—H···*A*	*D*—H	H···*A*	*D*···*A*	*D*—H···*A*
C2—H2···N3^i^	0.93	2.57	3.488 (2)	167
C9—H9A···O2^ii^	0.97	2.53	3.246 (3)	131
C12—H12···O2	0.93	2.44	2.924 (3)	112

Symmetry codes: (i) −*x*+1, −*y*+2, −*z*; (ii) −*x*+2, −*y*+1, −*z*.
